# Homicide impunity in Brazil between 2006 and 2016

**DOI:** 10.11606/s1518-8787.2020054002284

**Published:** 2020-11-27

**Authors:** Felipe Souza Nery, Paulo Nadanovsky

**Affiliations:** I Universidade Estadual de Feira de Santana Departamento de Saúde Feira de SantanaBA Brasil Universidade Estadual de Feira de Santana. Curso de Enfermagem. Departamento de Saúde. Feira de Santana, BA, Brasil; II Universidade do Estado do Rio de Janeiro Instituto de Medicina Social Departamento de Epidemiologia Rio de JaneiroRJ Brasil Universidade do Estado do Rio de Janeiro. Instituto de Medicina Social. Departamento de Epidemiologia. Rio de Janeiro, RJ, Brasil

**Keywords:** Homicide, legislation & jurisprudence, Violence, External Causes, Criminal Liability, Ecological Studies

## Abstract

**OBJECTIVE:**

To describe the level and temporal trends of homicide impunity in Brazil.

**METHODS:**

This is an ecological study that calculated two impunity indexes by dividing the total number of homicides committed in a 5-year period by the number of individuals arrested for murder (homicide impunity) or any other cause (general impunity) two years after this period. The Prais-Winsten linear regression model with serial autocorrelation correction was used to estimate the temporal trend of the impunity indexes.

**RESULTS:**

Between 2009 and 2014, 328,714 homicides were recorded in Brazil, but only 84,539 prisoners were serving sentences for this kind of crime in 2016. This shows that the number of homicides in Brazil exceeded in 244,175 the number of individuals in prisons for this crime. The impunity index ranged from 3.9 in 2006 to 3.3 in 2014. All states reached values above 1. Rio de Janeiro stood out negatively, with values above 20. São Paulo, Santa Catarina, and Distrito Federal showed the lowest impunity indexes for homicide, with values below 2. Eight states showed a downward trend in the overall impunity index.

**CONCLUSIONS:**

Most Brazilian states presented extremely high impunity indexes values. However, from 2010 to 2012, Brazilian society started to effectively combat impunity for serious violent crimes, including homicide. In São Paulo, this positive trend arose in the mid-1990s and that state currently shows impunity indexes values similar to those of developed countries.

## INTRODUCTION

Over 60 thousand homicides occur in Brazil every year, representing an annual rate of 32 murders per 100,000 inhabitants^1^. In developed countries, such rate is around 1 murder per 100,000 inhabitants^2^, revealing that Brazilian society is comparatively very violent.

Few doubt the central role played, historically, by the incarceration of criminals in reducing crime in developed countries ^3^. Arresting murder perpetrators has a direct and immediate effect, by preventing them from remaining free and committing crimes, and an indirect effect as, once free, a murderer (or serious violent crime perpetrator) who served sentence will think twice before committing another crime. In addition, people who get to know these perpetrators’ story will reconsider committing crimes^5^, as perceiving the probability and magnitude of punishment influences people’s decision on whether to perform criminal acts. From a “rational choice” perspective, short sentences and low probabilities of arrest reduce future costs of committing a crime^6^, while a high probability of punishment may prevent it^7^.

A study comparing countries from the Organization for Economic Cooperation and Development (OECD) and South America^4^ found impunity (i.e., low imprisonment for murder) and income inequality to be the main factors associated with high homicide rates. Another study, comparing Brazilian states, found impunity alone to be the main factor^10^. In São Paulo, increased incarceration was associated with the reduction in homicide rates^11^.

Among the 69 evaluated countries, Brazil was the seventh worst in terms of impunity – occupying a worse position than several Latin American countries, including Colombia, Paraguay, Chile, and Argentina^12^. Impunity is related to the low number of identified suspects, low crime clearance rate, time lapse from crime to punishment, low quality of investigation, small contingent of police officers per inhabitant, and the relatively small number of prisoners in countries with a large number of serious crimes^13^. We cannot state, relying solely on the absolute number of prisoners or the proportion of prisoners per inhabitants, that Brazil overincarcerates people. To do so, we must consider the number of individuals who commit serious crimes, such as murder, rape, robbery, and kidnapping. Incarceration rates are much higher in OECD countries than in South American countries: in OECD, for every 20 murders committed within the 10 years prior to the survey, 100 people were incarcerated for any crime. As for South American countries, for every 130 murders committed within the previous 10 years, only 100 people were incarcerated for any crime^4^.

Besides the utilitarian role of punishment in reducing crimes, one must also consider its psychological role, which is essential for the human being. The desire for punishing criminals arises from the feelings of vengeance, necessary for individuals to cooperate in society. In fact, such feelings are essential for the very development of cooperation^5^, being the key for social cohesion and the civilizing process^4^. Retaliation and vengeance are integral parts of our human psychology to the point that “our desire for justice essentially implies a desire for vengeance”^14^. That is, the moral basis of modern justice is the desire that the perpetrator suffer as much as the victim for the costs incurred, so that both are even^14^. For playing a role in inhibiting cheating, theft, aggression, and murder, this feeling evolved biologically. In other words, vengeance is an evolutionary adaptation that originated modern justice. “Vengeance is no disease: it is necessary for cooperation, preventing the ‘nice guy’ from being exploited”^5^. Thus, vengeance is one of the main moral emotions that are adaptations for cooperation^15^.

Given that punishment is helpful in combating criminal acts^5^; that it arises from the vengeance emotion (an essential part of the human psychology and the moral basis of the modern justice system^14^), and that vengeance is a human evolutionary adaptation necessary for social cohesion, civilization, and cooperation^15^, the impunity of serious violent crimes must be monitored to help reducing them, encouraging social cohesion and cooperation, as well as consolidating and advancing the civilizing process.

Considering that, this study sought to quantify homicide impunity (and, indirectly, the impunity of serious violent crimes in general) in the Brazilian states between 2006 and 2016.

## METHODS

This is a time series analysis of the 26 Brazilian states and the Federal District (DF) from 2002 to 2016. The study population comprised the population of each state and the DF. Homicide records were collected from the *Sistema de Informação sobre Mortalidade* (SIM – Mortality Information System) through the *Departamento de Informática do Sistema Único de Saúde* (DATASUS – Department of Informatics of the Brazilian Unified Health System). All violent causes of deaths classified between codes X85 and Y09 of the International Statistical Classification of Diseases and Related Health Problems, 10th revision (ICD-10), were included in the study. Homicides owing to legal interventions and operations of war committed by public security agents (classified in codes Y35 and Y36 of the ICD-10) were excluded; therefore, all deaths by homicide used to calculate impunity should entail at least one arrest for murder or its derivations (simple and qualified homicide and robbery aggravated by death).

The number of prisoners (tried or not) was obtained in *the Sistema Integrado de Informações Penitenciárias* (Infopen – Penitentiary Information Integrated System) through each state analytical report^a^. Our data included the total number of prisoners (for any cause, referenced as “prison population” in official documents) and the sum of prisoners for manslaughter, (art.121, § 3), simple (Art. 121, caput) and qualified (art. 121, § 2) homicide, and robbery aggravated by death (art. 157, § 3) reported in Infopen.

^a^Ministério da Justiça e Segurança Pública (BR), Departamento Penitenciário Nacional. SisDepen--Infopen: relatórios analíticos. Brasília, DF: DEPEN; s.d. [cited 2019 Jan 8]. Available from: http://depen.gov.br/DEPEN/depen/sisdepen/infopen/relatorios-analiticos

Homicide impunity index was calculated by the ratio between the number of homicides committed in five years and the number of individuals *incarcerated for murder* two years after this period. The 2010 impunity index, for example, was calculated by dividing the number of murders between 2006 and 2010 by the number of prison inmates doing time for homicide in 2012.

The global impunity index was calculated by the ratio between the number of homicides committed in five years and the number of *inmates doing time for any cause* two years after this period. That is, the 2010 global impunity index was calculated by dividing the number of murders between 2006 and 2010 by the number of prisoners for any cause in 2012. This index presupposes that the number of homicides is strongly and positively associated with the incidence of other forms of non-lethal violence, such as physical assaults, robbery, rapes, and kidnappings. This is justified by the fact that homicide tends to sporadically result from the escalating of these events, which occur much more frequently than murder itself. Thus, we consider murder as a *proxy* for these crimes.

These two indicators of impunity imply that murders and other violent crimes that occurred from 2006 to 2010 should result in a backlog of prisoners by 2012, consistent with the amount of these crimes. According to Article 121 of the Brazilian Penal Code, instituted by Decree-Law No. 2,848/1940, the sentence of imprisonment for murder, in its simplest form, ranges from 6 to 20 years, while that of qualified homicide may range from 12 to 30 years. As for robbery aggravated by death, sentence of imprisonment ranges from 20 to 30 years^16^. Other serious violent crimes also result in (or at least should) relatively long sentences (at least five years).

Considering that, if the number of murders from 2006 to 2010 is higher than the number of prisoners for this crime in 2012, we may assume there is impunity. Values above 1 would indicate homicide impunity, and its degree increases proportionally to the value. Similarly, values equal to 1 would indicate the lack of impunity within a federative unit, and values below 1 that the number of prisoners doing time for murder is greater than the actual number of murders. This last scenario enables at least three possible interpretations: the data is inaccurate; the amount of homicides with more than one perpetrator (partners in crime) is greater than that of multiple homicides with a single perpetrator (serial killers); or all murder perpetrators are being incarcerated and remaining in prison for more than five years, on average.

Global impunity index above 1 would indicate extreme impunity, as the number of prisoners would not represent the number of murders nor other serious violent crimes, such as rape, violent assault, robbery, and kidnapping. Values equal (or close) to 1 would indicate impunity, as the number of prisoners could represent the number of murders, but not other serious violent crimes. To represent both murders and other serious violent crimes, the global impunity index should present values well-below 1, given that murders represent but a small fraction of crimes that should result in incarceration.

We calculated homicide impunity index and global impunity index for the years of 2006, 2008, 2010, 2012, and 2014. The Prais-Winsten linear regression model with serial autocorrelation correction *was used to analyze the temporal trend of impunity indexes.* We adopted the methodological procedures described by Antunes and Cardoso^17^, including the percentage change (PC) calculation and its respective 95% confidence interval (95%CI). Our results are presented in the form of a forest plot, where the stationary trend-line (neither decreasing nor increasing) crosses the zero value. All analyses were performed in the *Stata* statistical program, version 12.

This study was based on the ethical principles of resolutions no. 466 of December 12, 2012, and no. 510 of April 7, 2016, of the National Health Council (NHC) – Ministry of Health (Brazil), which contemplate guidelines and norms regulating research involving human beings, exempting research from secondary source, freely accessible, free, and without subjects identification from ethical review^18,19^.

## RESULTS

Between 2009 and 2014, 328,714 homicides were recorded in Brazil, but only 84,539 prisoners were serving sentences for this kind of crime in 2016. This shows that the number of homicides in Brazil exceeded in 244,175 the number of prisoners for this crime. São Paulo recorded 100 prisoners for murder for every 134 homicides, whereas in Bahia this proportion was 1,742 homicides per 100 prisoners. The Northeast was the region with the worst scenario – with 122,542 homicides committed from 2009 to 2014, but only 19,494 people were in prison for this crime in 2016. For every 100 prisoners for any cause, Bahia recorded 222 murders, São Paulo 15, and Brazil 45 in the same period (Table 1).

**Table 1:** Number of homicides between 2009 and 2014 in relation to the number of prisoners doing time for murder and the number of prisoners doing time for any cause in 2016. Brazil and Brazilian regions and states.

Regions and states	Homicides	Prisoners for murder	Prisoners for any cause	Number of homicides for every 100 prisoners for murder in 2016	Number of homicides for every 100 prisoners for any cause in 2016*
				
	2009–2014	2016	2016	2009–2014	2009–2014
Brazil	328,714	84,539	726,712	389	45
North	35,311	7,610	50,285	464	70
Acre	1,156	228	5,364	507	22
Amapá	1,393	112	2,680	1244	52
Amazonas	7,065	1,526	11,390	463	62
Pará	19,656	3,445	14,212	571	138
Rondônia	3,113	1,672	10,832	186	29
Roraima	848	348	2,339	244	36
Tocantins	2,080	279	3,468	746	60
Northeast	122,542	19,494	129,742	629	94
Alagoas	12,483	2,087	6,957	598	179
Bahia	34,013	1,952	15,294	1742	222
Ceará	20,580	4,746	34,566	434	60
Maranhão	10,898	1,159	8,835	940	123
Paraíba	8,959	1,640	11,377	546	79
Pernambuco	20,705	5,056	34,556	410	60
Piauí	3,068	919	4,032	334	76
Rio Grande do Norte	6,835	606	8,809	1128	78
Sergipe	5,001	1,329	5,316	376	94
Midwest	30,574	9,109	61,161	336	50
Distrito Federal	5,195	4,099	15,194	127	34
Goiás	14,803	2,411	16,917	614	88
Mato Grosso	6,528	253	10,362	2,580	63
Mato Grosso do Sul	4,048	2,346	18,688	173	22
Southeast	101,756	39,967	378,047	255	27
Espírito Santo	10,342	3,563	19,413	290	53
Minas Gerais	25,631	9,512	68,354	269	37
Rio de Janeiro	29,748	-	50,219	-	59
São Paulo	36,035	26,892	240,061	134	15
South	38,531	8,359	107,040	461	36
Paraná	19,898	3,009	51,700	661	38
Rio Grande do Sul	13,745	2,668	33,868	515	41
Santa Catarina	4,888	2,682	21,472	182	23

* Including prisioners doing time for murder.

Source: Sistema de Informação sobre Mortalidade/Datasus and Infopen. Elaborated by the authors (2019).

In Brazil, the homicide impunity index ranged from 3.9 in 2006 to 3.3 in 2014 (Table 2), while global impunity index ranged from 0.54 to 0.38 (Table 3). We observed the highest homicide impunity indexes in Rio de Janeiro – where values were above 20 for all years with data on this index, – Bahia, Maranhão, and Alagoas, and, more recently, Amapá and Rio Grande do Norte (Table 2). Alagoas, Bahia, Maranhão, in the Northeast, and Pará, in the North, presented the highest global impunity indexes. In the Southeast, Rio de Janeiro presented the highest values and São Paulo the lowest (Table 3).

**Table 2:** Distribution of homicide impunity indexes*. Brazil and Brazilian regions and states, 2006–2014.

Regions and states	Years				

	2006	2008	2010	2012	2014
Brazil	3.9	3.9	3.2	3.8	3.3
North	3.6	3.5	4.1	5.5	4.0
Acre	1.1	0.8	1.2	3.3	4.4
Amapá	2.4	2.5	2.5	10.5	10.7
Amazonas	5.5	5.0	5.3	8.6	4.0
Pará	5.4	5.3	5.6	5.3	4.8
Rondônia	2.9	2.3	2.5	3.6	1.5
Roraima	1.9	1.7	2.4	1.9	2.1
Tocantins	2.2	3.5	3.4	15.1	6.4
Northeast	3.8	5.6	3.8	7.0	5.4
Alagoas	11.1	7.6	6.9	10.3	5.1
Bahia	6.1	8.6	11.5	17.7	14.7
Ceará	2.3	-	1.8	2.4	3.9
Maranhão	4.5	7.1	7.1	12.9	8.2
Paraíba	2.1	5.2	2.5	5.4	4.7
Pernambuco	4.0	3.6	2.9	10.2	3.3
Piauí	2.3	2.9	2.7	4.2	2.9
Rio Grande do Norte	2.7	2.9	3.6	11.6	10.0
Sergipe	4.4	4.3	3.7	4.8	3.3
Midwest	2.6	2.5	2.2	3.0	2.9
Distrito Federal	1.4	1.3	1.1	1.1	1.1
Goiás	4.3	3.8	4.2	8.9	5.4
Mato Grosso	2.5	3.0	2.6	7.9	21.9
Mato Grosso do Sul	2.9	2.2	1.9	1.6	1.4
Southeast	4.4	3.5	2.8	2.3	2.1
Espírito Santo	5.0	4.5	3.5	2.8	2.4
Minas Gerais	5.3	4.6	3.6	2.8	2.3
Rio de Janeiro	-	21.8	25.9	25.5	-
São Paulo	2.6	1.9	1.4	1.2	1.1
South	3.8	4.4	4.1	5.0	3.8
Paraná	3.5	4.4	4.5	6.6	5.4
Rio Grande do Sul	7.8	9.3	8.4	5.5	4.3
Santa Catarina	1.8	1.7	1.5	2.2	1.5

* Calculated by the ratio between the number of homicides in a five-year interval by the number of prisoners doing time for homicide two years after this period. Homicide impunity index should be ideally close to 1, indicating no impunity for murder.

Source: Sistema de Informação sobre Mortalidade/Datasus and Infopen. Elaborated by the authors (2019).

**Table 3:** Distribution of global impunity indexes*. Brazil and Brazilian regions and states, 2006–2014.

Regions and states	Years				

	2006	2008	2010	2012	2014
BRAZIL	0.54	0.49	0.46	0.42	0.38
North	0.62	0.62	0.65	0.52	0.60
Acre	0.23	0.18	0.21	0.19	0.19
Amapá	0.44	0.52	0.13	0.42	0.45
Amazonas	0.70	0.62	2.07	0.62	0.54
Pará	0.92	1.09	1.15	1.24	1.17
Rondônia	0.48	0.35	0.35	0.14	0.24
Roraima	0.34	0.30	0.32	0.36	0.31
Tocantins	0.53	0.50	0.52	0.28	0.52
Northeast	0.86	0.83	0.92	0.92	0.81
Alagoas	2.71	2.45	2.02	1.71	1.53
Bahia	0.88	0.95	1.74	1.75	1.87
Ceará	0.60	0.59	0.62	0.62	0.53
Maranhão	0.87	0.91	1.16	1.13	1.08
Paraíba	0.39	0.51	0.62	0.66	0.68
Pernambuco	1.10	0.92	0.73	0.70	0.48
Piauí	0.76	0.68	0.67	0.67	0.67
Rio Grande do Norte	0.48	0.41	0.47	0.59	0.69
Sergipe	0.84	0.76	0.80	0.75	0.82
Midwest	0.36	0.36	0.36	0.35	0.36
Distrito Federal	0.44	0.39	0.34	0.30	0.28
Goiás	0.68	0.66	0.72	0.69	0.76
Mato Grosso	0.42	0.39	0.44	0.49	0.53
Mato Grosso do Sul	0.26	0.31	0.28	0.25	0.18
Southeast	0.52	0.41	0.31	0.25	0.22
Espírito Santo	0.84	0.82	0.64	0.54	0.43
Minas Gerais	0.45	0.45	0.38	0.33	0.32
Rio de Janeiro	1.48	1.30	0.86	0.62	0.49
São Paulo	0.37	0.24	0.17	0.14	0.12
South	0.35	0.36	0.41	0.45	0.30
Paraná	0.37	0.43	0.54	0.62	0.31
Rio Grande do Sul	0.35	0.34	0.37	0.39	0.34
Santa Catarina	0.26	0.23	0.22	0.24	0.19

* Calculated by the ratio between the number of homicides in a five-year interval by the number of prisoners doing time for any cause two years after this period. Global impunity index should be ideally from 0.10 to 0.20, indicating little impunity for serious crimes (developed countries showed values between 0.10 and 0.20 in the early 2000s)^4^.

Source: Sistema de Informação sobre Mortalidade/Datasus and Infopen. Elaborated by the authors (2019).

**Figure 1:**
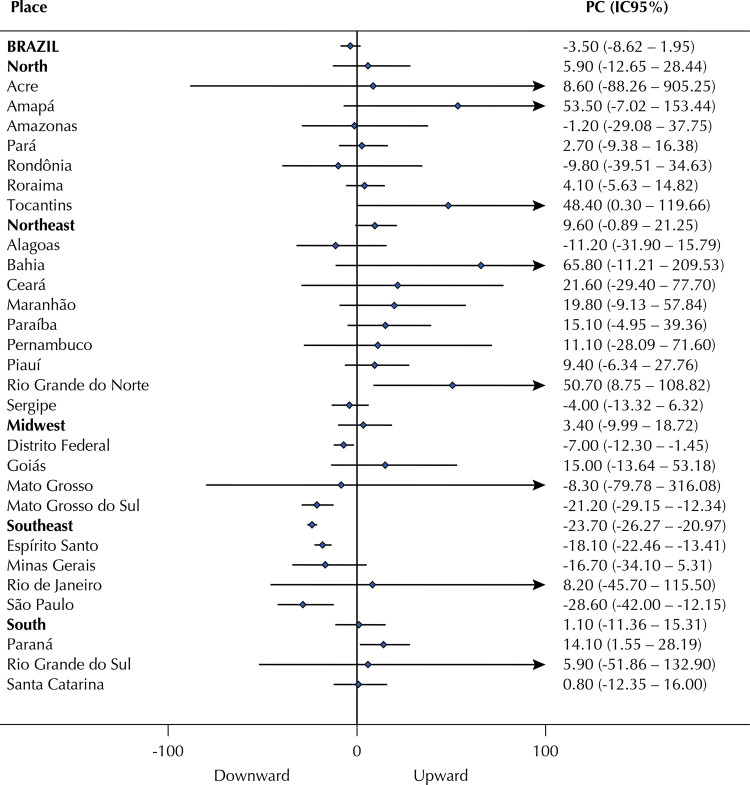
Percentage change (PC)* and 95% confidence interval of the homicide impunity index for every two years. Brazil and Brazilian regions and states, 2006–2014.

No state in the North, Northeast, and South regions showed a clear downward trend in homicide impunity index. In turn, DF and Mato Grosso do Sul, in the Midwest, and Espírito Santo and São Paulo, in the Southeast, showed a clear downward trend in this index. Thus, only four Brazilian states presented, with some confidence, a decreasing tendency for homicide impunity between 2006 and 2014. The percentage change in homicide impunity index was -28.6% (95%CI -42 – -12.2) in São Paulo, the highest reduction in the country (Figure 1).

* Prais-Winsten linear regression model with serial autocorrelation correction.

Source: Sistema de Informação sobre Mortalidade/Datasus and Infopen. Elaborated by the authors (2019).

**Figure 2:**
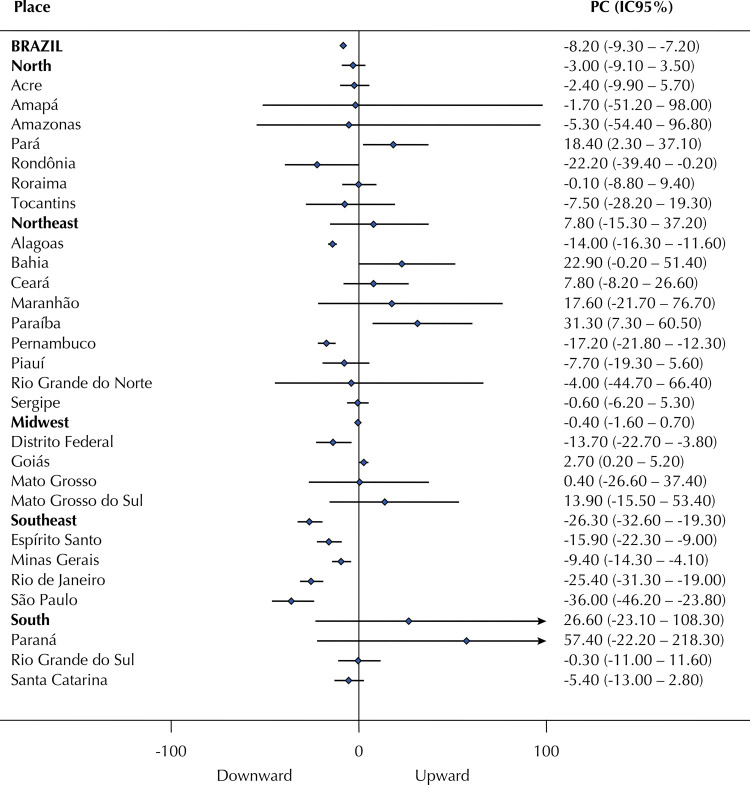
Percentage change (PC)* and 95% confidence interval of the global impunity index for every two years. Brazil and Brazilian regions and states, 2006–2014.

Rondônia, in the North, Alagoas and Pernambuco, in the Northeast, DF, in the Midwest, and all southeastern states showed a clear downward trend in the global impunity index. Except for the southern region, at least one state from each Brazilian region (totaling eight states) showed a decreasing tendency for global impunity between 2006 and 2014 (Figure 2).

* Prais-Winsten linear regression model with serial autocorrelation correction.

Source: Sistema de Informação sobre Mortalidade/Datasus and Infopen. Elaborated by the authors (2019).

## DISCUSSION

The global impunity index in Bahia, Alagoas, Pará, and Maranhão was so extreme that the number of murders between 2009 and 2014 was even higher than the number of prisoners doing time for any cause in 2016. That is, the number of imprisonments in these four states was so small in relation to the number of murders that it could not represent neither murders nor other serious violent crimes.

Although not as dramatic, global impunity levels were still significantly high in the other states – the number of prisoners for any cause was higher than the number of murders, but not enough to represent other serious violent crimes. All states showed very high homicide impunity indexes; for almost every four murders committed in Brazil between 2009 and 2014, there was only one person in prison for murder in 2016. Homicide impunity index must be ideally close to 1, indicating that one perpetrator was arrested for each murder recorded (given that one incarcerated person may be responsible for more than one murder, or that one murder may have been committed by more than one perpetrator). Our results confirm that Brazil is one of the world’s most violent countries, with a great risk of murder^2^ and a high level of impunity^12^.

This study shows, broadly and numerically, the outcomes of impunity mechanisms for serious violent crime in Brazil. As an example, the Violence Monitor reported that, among the 1,195 violent criminal acts that occurred in a random week of 2018 in Brazil, only 39% of the perpetrators were identified, and 2% received a judicial conviction within 316 days^20,21^.

Brazil, along with Venezuela and Colombia, has one of the worst clearance rates for homicide in the world. In fact, Latin American countries altogether feature in the hall of countries that are ineffective in identifying suspects of serious crimes and present difficulties in conducting an appropriate investigation. For every 100 murders, Asia identifies 151 suspects and punishes 48 perpetrators while American justice identifies only 53 suspects and punishes 24. European countries, such as Germany and Switzerland, show the best clearance rates, reaching approximately 95%^2,22^. In the US, this rate was 56% in 2010^23^.

In turn, 92% to 95% of murders in Brazil remain unsolved^24^. Until 2009, for example, the state of Ceará piled up 1,416 open homicides investigations; a decade later, only 27% of them lead to judicial complaints^25^. In Fortaleza, only 4% of murders committed in 2017 went to trial in the same year^26^. These findings reflect a scenario of injustice and indignation. In Ceará, residents of a violent neighborhood considered homicide impunity a serious crime whose perpetrator is the State itself^27^.

Conversely, São Paulo stood out positively in reducing impunity indexes: the extension of murder investigation period in the state was 7.5 years in 2009, decreasing to 2 years by 2015; São Paulo also established the first Department of Homicides and Protection of the Person (DHPP) in 1986, reformulated in the 2000s, which became a national reference for facilitating homicides clarification^21^.

This study has some limitations, such as the data quality. We found no data on the number of prisoners due to murder in 2008 and 2016 in Rio de Janeiro, preventing us from calculating the homicide impunity index for two moments. Moreover, Infopen data has not been updated recently, which impairs the conduct of research in the country due to underreporting^28^.

One limitation of our global impunity index was using homicide as an indicator of other serious violent crimes. Given the lack of more direct and accurate data on all serious violent crimes, we used values reported by developed countries (from OECD) in the early 2000s as a parameter, inferring that an ideal index would range from 0.10 to 0.20^4^. In the early 2000s, OECD countries reported an average of 18 homicides for every 100 prisoners for any cause (0.18)^4^ – a similar value to that found for the state of São Paulo in this study (0.15), but much lower than that for the whole country (0.45).

The indexes in our study probably underestimated the impunity of serious violent crimes in Brazil, such as murder, rape, robbery, and kidnapping, as some prison inmates computed in the calculation of our global impunity index did not even commit such criminal acts (for example, people convicted for minor crimes, or those who were not tried and could still be acquitted). We also analyzed only a five-year period of homicide records.

Impunity levels for serious violent crimes in Brazil differ from those of a modern, democratic society and a rule of law, such as those found in developed countries^4^. However, from the mid-1990s to 2005, São Paulo experienced a sharply rise in the incarceration rate with a subsequent reduction in homicide rate^11^ and, until the period analyzed in this study (2006-2016), the state was consolidated as the great positive exception in Brazil, showing a low impunity index, similar to that of modern developed societies.

It is unsettling and frustrating that, despite the positive examples from developed countries and São Paulo, the other Brazilian states have not shown a clear and consistent downward trend in homicide impunity indexes, besides still presenting high impunity levels for serious violent crimes.

Meanwhile, we found the first signs of a clear decreasing tendency for global impunity indexes between 2006 and 2014 in Southeast states, as well as in some states from other regions. Reductions in global impunity indexes imply reductions in homicide impunity. If such trend remains, intensifies, and extrapolates to other states, we anticipate a significant decrease in the number of homicides and serious violent crimes in Brazil for the coming years.

## CONCLUSION

Most Brazilian states presented extremely high impunity indexes values. São Paulo, Espírito Santo, Mato Grosso do Sul, and DF alone showed a clear downward trend in homicide impunity.

We expect the State, alongside civil society, to develop strategies for minimizing impunity of homicides and serious violent crimes in Brazil by quickly identifying suspects, investigating cases, and bringing to justice true perpetrators to ensure their sentences are properly served. This would not only incapacitate aggressors and inhibit further assaults, but also create an environment of more social cooperation, justice, and peace.

We found a reduction in the global impunity index in eight states spread across four Brazilian regions, positively indicating that from 2010 to 2012 the country began to effectively combat impunity for serious violent crimes, including homicide. In São Paulo, this positive trend arose in the mid-1990s and the state currently shows impunity indexes similar to those of developed countries.

## Footnotes

a Ministério da Justiça e Segurança Pública (BR), Departamento Penitenciário Nacional. SisDepen--Infopen: relatórios analíticos. Brasília, DF: DEPEN; s.d. [cited 2019 Jan 8]. Available from: http://depen.gov.br/DEPEN/depen/sisdepen/infopen/relatorios-analiticos
