# Supplementary material for: Homicide impunity in Brazil between 2006 and 2016
Source: Rev Saude Publica. 2020 Nov 27;54:144. doi: 10.11606/s1518-8787.2020054002284 (PMC7702386; doi:10.11606/s1518-8787.2020054002284)
Supplement: Translation

# Translation: A impunidade do homicídio no Brasil entre 2006 e 2016

**Authors:** Felipe Souza Nery, Paulo Nadanovsky

**Affiliations:** I Brasil Universidade Estadual de Feira de Santana. Curso de Enfermagem. Departamento de Saúde. Feira de Santana, BA, Brasil; II Brasil Universidade do Estado do Rio de Janeiro. Instituto de Medicina Social. Departamento de Epidemiologia. Rio de Janeiro, RJ, Brasil

**Keywords:** Homicídio, legislação & jurisprudência, Violência, Causas Externas, Responsabilidade Penal, Estudos Ecológicos

## Abstract

**OBJETIVO:**

Descrever o nível e a tendência temporal da impunidade do homicídio no Brasil.

**MÉTODOS:**

Trata-se de estudo ecológico no qual dois índices de impunidade foram calculados a partir do número total de homicídios em determinado período, de cinco anos, dividido pelo número de indivíduos na prisão por homicídio (impunidade do homicídio) ou por qualquer causa (impunidade geral) dois anos após o final desse período. O modelo de regressão linear com correção de autocorrelação serial de Prais-Winsten foi utilizado para estimar a tendência temporal dos índices de impunidade.

**RESULTADOS:**

No Brasil, entre 2009 e 2014, foram identificados 328.714 homicídios, contudo apenas 84.539 presos cumpriam pena por esse tipo de delito em 2016, revelando que houve 244.175 mais casos de homicídio no Brasil do que presos por esse crime. O índice de impunidade do homicídio variou de 3,9, em 2006, a 3,3 em 2014. Todos os estados apresentaram valores acima de 1. O Rio de Janeiro destacou-se negativamente, com valores acima de 20. Os menores índices de impunidade do homicídio foram encontrados nos estados de São Paulo, Santa Catarina e Distrito Federal, com valores abaixo de 2. Oito estados mostraram tendência de redução no índice de impunidade geral.

**CONCLUSÕES:**

A maioria dos estados brasileiros apresentou valores altíssimos nos índices de impunidade. No entanto, detectamos um sinal positivo de que a sociedade brasileira começou, a partir de 2010–2012, a combater de forma efetiva a impunidade dos crimes violentos graves, incluindo o homicídio. São Paulo iniciou essa tendência positiva em meados dos anos 1990 e apresenta atualmente índices de impunidade similares aos dos países desenvolvidos.

## INTRODUÇÃO

Anualmente mais de 60 mil homicídios ocorrem no Brasil, e a taxa anual desse crime é de 32 por 100 mil habitantes^1^. Países desenvolvidos, por sua vez, apresentam taxas de cerca de 1 homicídio por 100 mil habitantes^2^, revelando que a sociedade brasileira é comparativamente muito violenta.

Há poucas dúvidas quanto ao papel central, historicamente, do encarceramento de criminosos na redução do crime em países desenvolvidos^3^. A prisão do autor do homicídio tem um efeito direto e imediato, impedindo que ele continue livre e cometendo crimes; e também um efeito indireto: um autor de homicídio (ou de crime violento grave) que cumpriu pena pensará duas vezes antes de cometer outro crime quando estiver livre. Além disso, pessoas que conhecerem sua história também repensarão antes de cometer um delito^5^. A percepção da probabilidade e magnitude da punição influencia a decisão de cometer ou não um crime. Numa perspectiva de “escolha racional”, sentenças curtas e baixas probabilidades de detenção reduzem os custos futuros de cometer um delito^6^, enquanto uma alta probabilidade de punição pode evitá-lo^7^.

Impunidade (i.e., níveis baixos de encarceramento por homicídio) e desigualdade de renda foram os principais fatores associados a altas taxas de homicídio em um estudo que comparou os países da Organização para a Cooperação e Desenvolvimento Econômico (OCDE) e da América do Sul^4^. Em outro estudo, que comparou os estados brasileiros, a impunidade foi isoladamente o principal fator encontrado^10^. O aumento do encarceramento de criminosos foi associado à redução na taxa de homicídios em São Paulo^11^.

O Brasil foi considerado o sétimo país com mais impunidade dentre os 69 pesquisados, resultado pior que o de diversos países da América Latina, incluindo Colômbia, Paraguai, Chile e Argentina^12^. A impunidade está relacionada ao baixo número de suspeitos identificados, à baixa taxa de esclarecimento do crime, ao lapso temporal entre o crime e a punição, à baixa qualidade da investigação, ao pequeno contingente de policiais por habitantes, e ao número relativamente pequeno de presos em países com número grande de crimes graves^13^. É enganoso afirmar, com base somente no número absoluto de presos ou na proporção de presos por habitantes, que o Brasil encarcera pessoas excessivamente. Para avaliar se o país prende pessoas demais é necessário considerar também o número de indivíduos que cometem crimes graves, tais como homicídio, estupro, assalto e sequestro. Há uma grande diferença nas taxas de encarceramento de criminosos entre os países da OCDE e da América do Sul, com valores muito maiores naqueles da OCDE, onde, para cada 20 homicídios nos 10 anos anteriores ao da pesquisa, havia 100 pessoas na prisão por qualquer crime. Já nos países da América do Sul, para cada 130 homicídios nos 10 anos precedentes, havia somente 100 pessoas presas por qualquer crime^4^.

Além do papel utilitário da punição de criminosos para a redução do crime, deve-se considerar também sua função psicológica, essencial para o ser humano. O desejo de punição dos criminosos é fruto da emoção da vingança, que é necessária à cooperação entre indivíduos na sociedade. Na realidade, essa emoção é necessária à própria evolução da cooperação^5^ e, por isso, é chave para a coesão social e o processo civilizatório^4^. A retaliação e a vingança são partes constitutivas de nossa psicologia humana, tanto que “o nosso desejo por justiça fundamentalmente implica um desejo por vingança”^14^. Em outras palavras, a base moral da justiça moderna é o desejo de que o agressor sofra pelo que fez tanto quanto a vítima sofreu, de forma que ele e a vítima fiquem quites^14^. Esse sentimento evoluiu biologicamente, pois teve a função de inibir a trapaça, o roubo, a agressão e o assassinato, isto é, a vingança é uma adaptação evolutiva que deu origem à justiça moderna. “Vingança não é doença alguma: ela é necessária para a cooperação, evitando que o ‘cara legal’ seja explorado”^5^. A vingança é, assim, uma das principais emoções morais, que são adaptações para a cooperação^15^.

Dado que a punição dos criminosos é útil no combate ao crime^5^; que é fruto da emoção da vingança, sendo esta uma parte essencial da psicologia humana e a justificativa moral do sistema de justiça moderno^14^; e que a vingança é uma adaptação evolutiva humana necessária para a coesão social, o processo civilizatório e a cooperação entre as pessoas^15^, é importante monitorar a impunidade de crimes violentos graves na sociedade para ajudar a reduzi-los, a fim de estimular a coesão e a cooperação sociais, além de consolidar e fazer avançar o processo civilizatório.

Dessa forma, neste estudo o objetivo foi quantificar a impunidade do homicídio (e, indiretamente, de crimes violentos graves de forma geral) entre 2006 e 2016 nos estados brasileiros.

## MÉTODOS

Realizamos um estudo de série temporal cujas unidades de análise foram os 26 estados brasileiros e o Distrito Federal (DF), no período de 2002 a 2016. A população do estudo foi composta pela população de cada estado brasileiro e do DF. Os registros de homicídios foram obtidos no Sistema de Informação sobre Mortalidade (SIM), acessados a partir do Departamento de Informática do Sistema Único de Saúde (DataSUS). Consideramos as mortes violentas classificadas entre os códigos X85 e Y09 da Classificação Estatística Internacional de Doenças e Problemas Relacionados à Saúde, 10ª revisão (CID-10). Não incluímos neste estudo os homicídios em intervenções legais e operações de guerra que foram cometidos por agentes da segurança pública, classificados nos códigos Y35 e Y36 da CID-10. Assim, todos os óbitos por homicídio considerados para os cálculos da impunidade deveriam gerar ao menos uma prisão por homicídio ou suas derivações, como homicídio doloso simples, qualificado e latrocínio.

O número de presos (julgados ou não) foi obtido no Sistema Integrado de Informações Penitenciárias (Infopen), acessando-se os relatórios analíticos de cada estado^a^. Consideramos a quantidade total de presos (i.e., o total de presos por qualquer causa, referido em documentos oficiais como “população carcerária”) e a soma do número de presos por homicídio culposo (art. 121, § 3º), simples (art. 121, caput), qualificado (art. 121, § 2°) e latrocínio (art. 157, § 3°) registrados no Infopen.

^a^Ministério da Justiça e Segurança Pública (BR), Departamento Penitenciário Nacional. SisDepen--Infopen: relatórios analíticos. Brasília, DF: DEPEN; s.d. [citado 8 jan 2019]. Disponível em: http://depen.gov.br/DEPEN/depen/sisdepen/infopen/relatorios-analiticos

O índice de impunidade do homicídio foi obtido pela razão entre o número de homicídios ocorridos num intervalo de cinco anos e o número de *presos por homicídio* dois anos após o final do período considerado. Assim, o índice de impunidade do homicídio de 2010, por exemplo, foi calculado dividindo-se o número de homicídios ocorridos entre 2006 e 2010 pelo número de presos por homicídio em 2012.

O índice de impunidade geral foi obtido pela razão entre o número de homicídios ocorridos num intervalo de cinco anos e o número de *presos por qualquer causa* dois anos após o final do período considerado. Assim, o índice de impunidade geral de 2010, por exemplo, foi calculado dividindo-se o número de homicídios ocorridos entre 2006 e 2010 pelo número de presos por qualquer causa em 2012. Esse índice pressupõe que a quantidade de homicídios está forte e positivamente associada à frequência de outras formas de violência não letais, como agressões físicas, assaltos, estupros e sequestros, pois geralmente o homicídio é um resultado esporádico da escalada desses eventos, que são bem mais frequentes do que o próprio assassinato. O homicídio, portanto, é aqui tomado como *proxy* desses outros crimes.

Esses dois indicadores de impunidade pressupõem que os homicídios e outros crimes violentos graves ocorridos entre 2006 e 2010 devem gerar um acúmulo de presos em 2012, compatível com o número desses delitos. Enfatiza-se que, segundo o art. 121 do Código Penal brasileiro, instituído pelo Decreto-Lei nº 2.848/1940, a pena de reclusão para o homicídio, em sua forma mais simples, varia de 6 a 20 anos, enquanto a do homicídio qualificado pode variar de 12 a 30 anos. Já em caso de latrocínio, a pena de reclusão varia entre 20 e 30 anos^16^. Outros crimes violentos graves também redundam (ou deveriam redundar) em penas de reclusão relativamente longas (pelo menos de cinco anos).

Dessa forma, caso o número de homicídios ocorridos entre 2006 e 2010 seja superior ao número de presos por esse crime em 2012, infere-se que há impunidade. Assim, valores acima de 1 indicariam impunidade do homicídio, sendo o seu grau tão maior quanto mais estiver afastado positivamente de 1. Partindo-se da mesma lógica, valores iguais a 1 seriam encontrados em unidades da Federação que não apresentam impunidade. Valores abaixo de 1 significariam que houve um número maior de presos por homicídio do que de homicídios, de fato. Neste caso, há pelo menos três interpretações possíveis: os dados estão errados; ou houve maior quantidade de homicídios com mais de um autor (parceiros no crime) do que de múltiplos homicídios de um mesmo autor (matadores seriais); ou todos os autores de homicídio estão sendo encarcerados e permanecendo na prisão por períodos, em média, mais longos do que cinco anos.

Valores do índice de impunidade geral maiores que 1 indicariam impunidade extrema, pois o número de presos não poderia dar conta dos homicídios, muito menos dos outros crimes violentos graves, tais como estupro, agressão violenta, assalto e sequestro. Valores iguais a 1 (ou não muito menores do que 1) indicariam impunidade, pois o número de presos poderia dar conta apenas dos homicídios, mas não de outros crimes violentos graves. Para abarcar homicídios e outros crimes violentos graves, o índice de impunidade geral deveria ter valores bem menores que 1, já que os homicídios são uma fração bem pequena de todos os crimes que deveriam redundar em reclusão.

Foi possível calcular o índice de impunidade do homicídio e o índice de impunidade geral dos anos de 2006, 2008, 2010, 2012 e 2014. Para a análise de tendência temporal dos índices de impunidade utilizamos o modelo de regressão linear com correção de autocorrelação serial de *Prais-Winsten.* Adotamos os procedimentos metodológicos descritos por Antunes e Cardoso^17^, incluindo o cálculo da mudança percentual (MP) e seu respectivo intervalo de confiança de 95% (IC95%). Esses resultados foram apresentados na forma de gráfico de floresta, em que a linha da tendência estacionária (i.e., nem decrescente nem crescente) passa pelo valor zero. Utilizamos o programa estatístico *Stata,* versão 12.

Este estudo foi baseado nos princípios éticos das Resoluções nº 466, de 12 de dezembro de 2012, e nº 510, de 7 de abril de 2016, do Conselho Nacional de Saúde (CNS) – Ministério da Saúde (Brasil), que contemplam as diretrizes e normas regulamentadoras de pesquisas envolvendo seres humanos, isentando de apreciação ética estudos de fonte secundária, acessíveis de forma livre, gratuita e sem identificação dos sujeitos^18,19^.

## RESULTADOS

No Brasil, entre 2009 e 2014, foram identificados 328.714 homicídios, contudo apenas 84.539 presos cumpriam pena por esse tipo de delito em 2016, revelando que houve 244.175 mais casos de homicídio do que presos por esse crime. Em São Paulo, para cada 134 homicídios havia 100 presos por esse delito, e na Bahia essa proporção era de 1.742 homicídios para 100 presos. O Nordeste foi a região com o pior cenário, somando 122.542 homicídios no período de 2009 a 2014 e apenas 19.494 presos por esse crime em 2016. No mesmo período, na Bahia, para cada 222 homicídios havia 100 presos por qualquer causa; em São Paulo, para cada 15 homicídios havia 100 presos; e, no Brasil, para cada 45 havia 100 (Tabela 1).

**Tabela 1:** Número de homicídios entre 2009 e 2014 em relação ao número de presos por homicídio e número de presos por qualquer causa em 2016. Brasil, regiões e estados brasileiros.

Regiões e estados	Homicídios	Presos por homicídio	Presos por qualquer causa	Homicídios para cada 100 presos por homicídio em 2016	Homicídios para cada 100 presos por qualquer causa em 2016*
				
	2009–2014	2016	2016	2009–2014	2009–2014
Brasil	328.714	84.539	726.712	389	45
Norte	35.311	7.610	50.285	464	70
Acre	1.156	228	5.364	507	22
Amapá	1.393	112	2.680	1244	52
Amazonas	7.065	1.526	11.390	463	62
Pará	19.656	3.445	14.212	571	138
Rondônia	3.113	1.672	10.832	186	29
Roraima	848	348	2.339	244	36
Tocantins	2.080	279	3.468	746	60
Nordeste	122.542	19.494	129.742	629	94
Alagoas	12.483	2.087	6.957	598	179
Bahia	34.013	1.952	15.294	1742	222
Ceará	20.580	4.746	34.566	434	60
Maranhão	10.898	1.159	8.835	940	123
Paraíba	8.959	1.640	11.377	546	79
Pernambuco	20.705	5.056	34.556	410	60
Piauí	3.068	919	4.032	334	76
Rio Grande do Norte	6.835	606	8.809	1128	78
Sergipe	5.001	1.329	5.316	376	94
Centro-Oeste	30.574	9.109	61.161	336	50
Distrito Federal	5.195	4.099	15.194	127	34
Goiás	14.803	2.411	16.917	614	88
Mato Grosso	6.528	253	10.362	2.580	63
Mato Grosso do Sul	4.048	2.346	18.688	173	22
Sudeste	101.756	39.967	378.047	255	27
Espírito Santo	10.342	3.563	19.413	290	53
Minas Gerais	25.631	9.512	68.354	269	37
Rio de Janeiro	29.748	-	50.219	-	59
São Paulo	36.035	26.892	240.061	134	15
Sul	38.531	8.359	107.040	461	36
Paraná	19.898	3.009	51.700	661	38
Rio Grande do Sul	13.745	2.668	33.868	515	41
Santa Catarina	4.888	2.682	21.472	182	23

* Incluindo presos por homicídio.

Fonte: Sistema de Informação sobre Mortalidade/Datasus e Infopen. Elaboração dos autores (2019).

No Brasil, o índice de impunidade do homicídio variou de 3,9, em 2006, a 3,3 em 2014 (Tabela 2), e o de impunidade geral variou de 0,54 a 0,38 (Tabela 3). Destacaram-se com os maiores índices de impunidade do homicídio o Rio de Janeiro – para todos os anos em que havia informação, esse índice apresentou valor acima de 20 –, Bahia, Maranhão e Alagoas, além de Amapá e Rio Grande do Norte, mais recentemente (Tabela 2). Os estados com maiores índices de impunidade geral foram Alagoas, Bahia e Maranhão, no Nordeste, e Pará, no Norte. O Rio de Janeiro obteve valores mais altos do que seus pares no Sudeste, e São Paulo os valores mais baixos (Tabela 3).

**Tabela 2:** Distribuição dos índices de impunidade do homicídio*. Brasil, regiões e estados brasileiros, 2006–2014.

Regiões e estados	Anos				

	2006	2008	2010	2012	2014
Brasil	3,9	3,9	3,2	3,8	3,3
Norte	3,6	3,5	4,1	5,5	4,0
Acre	1,1	0,8	1,2	3,3	4,4
Amapá	2,4	2,5	2,5	10,5	10,7
Amazonas	5,5	5,0	5,3	8,6	4,0
Pará	5,4	5,3	5,6	5,3	4,8
Rondônia	2,9	2,3	2,5	3,6	1,5
Roraima	1,9	1,7	2,4	1,9	2,1
Tocantins	2,2	3,5	3,4	15,1	6,4
Nordeste	3,8	5,6	3,8	7,0	5,4
Alagoas	11,1	7,6	6,9	10,3	5,1
Bahia	6,1	8,6	11,5	17,7	14,7
Ceará	2,3	-	1,8	2,4	3,9
Maranhão	4,5	7,1	7,1	12,9	8,2
Paraíba	2,1	5,2	2,5	5,4	4,7
Pernambuco	4,0	3,6	2,9	10,2	3,3
Piauí	2,3	2,9	2,7	4,2	2,9
Rio Grande do Norte	2,7	2,9	3,6	11,6	10,0
Sergipe	4,4	4,3	3,7	4,8	3,3
Centro-Oeste	2,6	2,5	2,2	3,0	2,9
Distrito Federal	1,4	1,3	1,1	1,1	1,1
Goiás	4,3	3,8	4,2	8,9	5,4
Mato Grosso	2,5	3,0	2,6	7,9	21,9
Mato Grosso do Sul	2,9	2,2	1,9	1,6	1,4
Sudeste	4,4	3,5	2,8	2,3	2,1
Espírito Santo	5,0	4,5	3,5	2,8	2,4
Minas Gerais	5,3	4,6	3,6	2,8	2,3
Rio de Janeiro	-	21,8	25,9	25,5	-
São Paulo	2,6	1,9	1,4	1,2	1,1
Sul	3,8	4,4	4,1	5,0	3,8
Paraná	3,5	4,4	4,5	6,6	5,4
Rio Grande do Sul	7,8	9,3	8,4	5,5	4,3
Santa Catarina	1,8	1,7	1,5	2,2	1,5

* Calculado por meio da razão do número de homicídios em um intervalo de cinco anos, pelo número de presos por homicídio, dois anos após o final do período considerado. O desejado é um valor próximo de 1,0, que indica ausência de impunidade do homicídio.

Fonte: Sistema de Informação sobre Mortalidade/Datasus e Infopen. Elaboração dos autores (2019).

**Tabela 3:** Distribuição dos índices de impunidade geral*. Brasil, regiões e estados brasileiros, 2006–2014.

Regiões e estados	Anos				

	2006	2008	2010	2012	2014
BRASIL	0,54	0,49	0,46	0,42	0,38
Norte	0,62	0,62	0,65	0,52	0,60
Acre	0,23	0,18	0,21	0,19	0,19
Amapá	0,44	0,52	0,13	0,42	0,45
Amazonas	0,70	0,62	2,07	0,62	0,54
Pará	0,92	1,09	1,15	1,24	1,17
Rondônia	0,48	0,35	0,35	0,14	0,24
Roraima	0,34	0,30	0,32	0,36	0,31
Tocantins	0,53	0,50	0,52	0,28	0,52
Nordeste	0,86	0,83	0,92	0,92	0,81
Alagoas	2,71	2,45	2,02	1,71	1,53
Bahia	0,88	0,95	1,74	1,75	1,87
Ceará	0,60	0,59	0,62	0,62	0,53
Maranhão	0,87	0,91	1,16	1,13	1,08
Paraíba	0,39	0,51	0,62	0,66	0,68
Pernambuco	1,10	0,92	0,73	0,70	0,48
Piauí	0,76	0,68	0,67	0,67	0,67
Rio Grande do Norte	0,48	0,41	0,47	0,59	0,69
Sergipe	0,84	0,76	0,80	0,75	0,82
Centro-Oeste	0,36	0,36	0,36	0,35	0,36
Distrito Federal	0,44	0,39	0,34	0,30	0,28
Goiás	0,68	0,66	0,72	0,69	0,76
Mato Grosso	0,42	0,39	0,44	0,49	0,53
Mato Grosso do Sul	0,26	0,31	0,28	0,25	0,18
Sudeste	0,52	0,41	0,31	0,25	0,22
Espírito Santo	0,84	0,82	0,64	0,54	0,43
Minas Gerais	0,45	0,45	0,38	0,33	0,32
Rio de Janeiro	1,48	1,30	0,86	0,62	0,49
São Paulo	0,37	0,24	0,17	0,14	0,12
Sul	0,35	0,36	0,41	0,45	0,30
Paraná	0,37	0,43	0,54	0,62	0,31
Rio Grande do Sul	0,35	0,34	0,37	0,39	0,34
Santa Catarina	0,26	0,23	0,22	0,24	0,19

* Calculado por meio da razão do número de homicídios em um intervalo de cinco anos, pelo número de presos por qualquer causa, dois anos após o final do período considerado. O desejado é um valor próximo de 0,10 a 0,20, que indica pouca impunidade de crimes graves (em países desenvolvidos foram encontrados valores entre 0,10 e 0,20 no início dos anos 2000)^4^.

Fonte: Sistema de Informação sobre Mortalidade/Datasus e Infopen. Elaboração dos autores (2019).

Nenhum estado nas regiões Norte, Nordeste e Sul mostrou clara tendência decrescente no índice de impunidade do homicídio. Já o DF e Mato Grosso do Sul, na região Centro-Oeste, e Espírito Santo e São Paulo, na região Sudeste, tiveram clara tendência decrescente nesse índice. Apenas quatro estados brasileiros mostraram, portanto, com alguma segurança, uma tendência de redução na impunidade do homicídio entre 2006 e 2014. São Paulo apresentou mudança percentual de -28,6% (IC95% -42 – -12,2) no índice de impunidade do homicídio, sendo o estado com a maior redução percentual (Figura 1).

**Figura 1:** Mudança percentual (MP)* e intervalo de confiança de 95%, para cada dois anos, do índice de impunidade do homicídio. Brasil, regiões e estados brasileiros, 2006–2014.

* Modelo de regressão linear com correção de autocorrelação serial de *Prais-Winsten*.

Fonte: Sistema de Informação sobre Mortalidade/Datasus e Infopen. Elaboração dos autores (2019).

Rondônia, no Norte; Alagoas e Pernambuco, no Nordeste; DF, no Centro-Oeste; e todos os estados do Sudeste apresentaram clara tendência decrescente no índice de impunidade geral. Portanto, com exceção da região Sul, houve pelo menos um estado em cada região do Brasil que mostrou, com alguma segurança, uma tendência de redução na impunidade geral entre 2006 e 2014; ao todo, oito estados apresentaram essa tendência (Figura 2).

**Figura 2:** Mudança percentual (MP)* e intervalo de confiança de 95%, para cada dois anos, do índice de *impunidade geral*. Brasil, regiões e estados brasileiros, 2006–2014.

* Modelo de regressão linear com correção de autocorrelação serial de *Prais-Winsten*.

Fonte: Sistema de Informação sobre Mortalidade/Datasus e Infopen. Elaboração dos autores (2019).

## DISCUSSÃO

Bahia, Alagoas, Pará e Maranhão tiveram um índice de impunidade geral tão extremo que o número de homicídios entre 2009 e 2014 foi maior do que o número de presos por *qualquer causa* em 2016. Ou seja, o número de presos nesses quatro estados foi tão pequeno em relação ao número de homicídios que não poderia ter dado conta nem dos assassinatos cometidos, muito menos dos outros crimes violentos graves.

Nos outros estados, os níveis de impunidade geral não foram tão dramáticos, mas ainda revelaram níveis altíssimos: o número de presos por qualquer causa foi maior do que o número de homicídios, mas não o suficiente para abarcar também os outros crimes violentos graves. Além disso, todos os estados apresentaram índices de impunidade do homicídio muito altos; no Brasil, para quase quatro homicídios cometidos entre 2009 e 2014, houve apenas um preso por esse crime em 2016. O desejável é que o índice de impunidade do homicídio apresente valores próximos de 1, ou seja, que haja um preso por homicídio para cada assassinato registrado (dado que, por um lado, um preso pode ter sido responsável por mais de um homicídio e, por outro lado, um mesmo homicídio pode ter sido cometido por mais de um autor). Nossos resultados ratificaram que o Brasil é um dos locais mais violentos do mundo, com alto risco de homicídio^2^ e alto grau de impunidade^12^.

Neste estudo demonstramos de forma ampla e numérica os desfechos dos mecanismos da impunidade do crime violento grave no Brasil. Por exemplo, o *Monitor da violência* relatou que em 2018, dentre os 1.195 crimes violentos ocorridos em uma semana aleatória no país, apenas 39% dos agressores foram identificados e 2% tiveram alguma condenação no prazo de 316 dias estabelecido pelo Judiciário^20,21^.

O Brasil tem uma das piores taxas de esclarecimento de homicídio, juntamente da Venezuela e da Colômbia. Aliás, os Estados da América Latina como um todo se destacam no *hall* de países ineficazes na identificação de suspeitos de crimes graves e apresentam dificuldades em conduzir uma investigação adequada. Enquanto na Ásia, para cada 100 homicídios, 151 suspeitos são identificados e, destes, 48 agressores são punidos, nas Américas a Justiça é capaz de identificar apenas 53 suspeitos e de punir 24. Encontramos os melhores resultados na Europa, em países como Alemanha e Suíça, que apresentaram taxas de esclarecimento dos homicídios próximas a 95%^2,22^. Nos EUA, em 2010, essa taxa foi de 56%^23^.

No Brasil, 92% a 95% dos homicídios não são solucionados^24^. O estado do Ceará, por exemplo, até 2007 apresentava um acúmulo de 1.416 inquéritos abertos sobre homicídios; porém, uma década depois, apenas 27% deles resultaram em denúncias à Justiça^25^. Apenas 4% dos homicídios em Fortaleza em 2017 foram a julgamento no mesmo ano^26^. Esses achados refletem um cenário de injustiça e indignação; no Ceará, moradores de um bairro violento consideraram a impunidade do homicídio um grave crime cometido pelo próprio Estado^27^.

Por outro lado, São Paulo se destacou positivamente na redução nos índices de impunidade. Em 2009, esse estado demorava 7,5 anos para concluir um inquérito de homicídio e, em 2015, esse tempo foi reduzido para 2 anos. Ademais, o estado implantou em 1986 o primeiro Departamento de Homicídios e Proteção à Pessoa (DHPP), reformulado nos anos 2000, que se tornou referência nacional por facilitar o esclarecimento de homicídios^21^.

Uma das limitações deste estudo reside na qualidade dos dados. No Rio de Janeiro, por exemplo, não havia informação sobre o número de presos por homicídio em 2008 e 2016, o que impediu o cálculo do índice de impunidade do homicídio em dois momentos. Além disso, não houve atualização recente dos dados do Infopen. A subnotificação prejudica a condução de pesquisas no país^28^.

Uma limitação do nosso índice de impunidade geral foi utilizar o homicídio como indicador dos outros tipos de crimes violentos graves. Na ausência de dados mais diretos e acurados sobre todos os crimes violentos graves, tomamos como parâmetro os valores encontrados nos países desenvolvidos (da OCDE) no início dos anos 2000, inferindo que o valor desejado para esse índice seria de 0,10 a 0,20^4^. Houve em média 18 homicídios para cada 100 presos por qualquer causa nos países da OCDE no início dos anos 2000 (índice de 0,18)^4^ – resultado similar ao valor que encontramos neste estudo para o estado de São Paulo (0,15), mas muito melhor do que o valor encontrado para o país todo (0,45).

Os índices deste estudo provavelmente subestimaram a impunidade de crimes violentos graves no Brasil, incluindo homicídio, estupro, assalto e sequestro, pois havia pessoas presas que não cometeram esse tipo de crime e, mesmo assim, entraram no cálculo do nosso índice de impunidade geral (por exemplo, pessoas condenadas por crimes menores, ou que não foram julgadas e poderiam ainda ser inocentadas). Além disso, utilizamos um período de apenas cinco anos de registros dos homicídios.

Os níveis de impunidade de crimes violentos graves no Brasil não são compatíveis com os de uma sociedade moderna, democrática e de um Estado de direito, como os encontrados em países desenvolvidos^4^. Contudo, há uma exceção: desde meados da década de 1990 até 2005, quando houve um grande aumento na taxa de encarceramento, com subsequente redução na taxa de homicídios^11^, e até o período analisado neste estudo (2006–2016), São Paulo se consolidou como a grande exceção positiva no Brasil, apresentando índice de impunidade baixo, similar ao das sociedades desenvolvidas modernas.

É preocupante e frustrante que, mesmo diante dos exemplos positivos dos países desenvolvidos e de São Paulo, os outros estados brasileiros não tenham mostrado sinais claros e consistentes de redução nos índices de impunidade do homicídio e ainda apresentem níveis tão altos de impunidade de crimes violentos graves.

Pelo lado positivo, encontramos os primeiros indicativos de que ao menos nos estados do Sudeste e em alguns estados de outras regiões o índice de impunidade geral mostrou sinais claros de redução entre 2006 e 2014. Reduções no índice de impunidade geral embutem reduções na impunidade do homicídio. Se essa tendência permanecer, for intensificada e estendida aos outros estados, prevemos reduções significativas nos homicídios e crimes violentos graves no Brasil nos próximos anos.

## CONCLUSÃO

A maioria dos estados brasileiros apresentou valores altíssimos nos índices de impunidade. Somente São Paulo, Espírito Santo, Mato Grosso do Sul e DF obtiveram dados convincentes de redução na impunidade do homicídio.

Espera-se que o Estado, juntamente com a sociedade civil, crie estratégias para minimizar a impunidade do homicídio e do crime violento grave no Brasil, identificando suspeitos rapidamente, investigando cada caso e levando ao conhecimento da Justiça o verdadeiro perpetrador do crime para que este possa cumprir devidamente sua pena. Assim, além de incapacitar os agressores e inibir novas agressões, criar-se-á um ambiente de mais cooperação social, justiça e paz.

A redução no índice de impunidade geral em oito estados espalhados em quatro regiões do país é um sinal positivo de que a sociedade brasileira começou, a partir de 2010–2012, a combater de forma efetiva a impunidade dos crimes violentos graves, incluindo o homicídio. São Paulo iniciou essa tendência positiva em meados dos anos 1990 e apresenta atualmente índices de impunidade similares aos de países desenvolvidos.

## Footnotes

a Ministério da Justiça e Segurança Pública (BR), Departamento Penitenciário Nacional. SisDepen--Infopen: relatórios analíticos. Brasília, DF: DEPEN; s.d. [citado 8 jan 2019]. Disponível em: http://depen.gov.br/DEPEN/depen/sisdepen/infopen/relatorios-analiticos
